# Benzyl Benzoate Isolation from *Acridocarpus smeathmannii* (DC.) Guill. & Perr Roots and Its Bioactivity on Human Prostate Smooth Muscle Contractions

**DOI:** 10.3390/ph18050687

**Published:** 2025-05-06

**Authors:** Oluwafemi Ezekiel Kale, Iskander Rauanov, Claudia Huber, Alexander Tamalunas, Christian G. Stief, Wolfgang Eisenreich, Martin Hennenberg

**Affiliations:** 1 Department of Pharmacology and Therapeutics, Faculty of Basic Medical Sciences, Olabisi Onabanjo University, Sagamu Campus, Ago-Iwoye 2001, Ogun State, Nigeria; 2 Bavarian NMR Center—Structural Membrane Biochemistry, Department of Chemistry, Technical University of Munich, 85748 Garching, Germany; iskander.rauanov@tum.de (I.R.); claudia.huber@tum.de (C.H.); wolfgang.eisenreich@mytum.de (W.E.); 3 Department of Urology, LMU University Hospital, LMU Munich, 80336 Munich, Germany; alexander.tamalunas@med.uni-muenchen.de (A.T.); christian.stief@med.uni-muenchen.de (C.G.S.); martin.hennenberg@med.uni-muenchen.de (M.H.)

**Keywords:** bioactivity-guided study, natural benzyl benzoate, *Acridocarpus smeathmannii*, prostate smooth muscle, drug discovery

## Abstract

**Background/Objectives:** This study is the first report on isolating a natural benzyl benzoate (nBB) from *Acridocarpus smeathmannii* (DC.) Guill. & Perr roots. **Methods**: The structure was verified using GC-MS, HPLC-UV-VIS, and two-dimensional NMR. Since it is known for its vasodilatory and anti-spasmolytic actions, we investigated the biological effects of nBB on human prostate smooth tissue (rPx) obtained from a radical prostatectomy. For this purpose, rPx was incubated with nBB (0.05, 0.25, or 0.5 µM) in an organ bath, and then cumulative concentration–response curves were constructed for adrenergic agonists and electrical field stimulation (EFS). **Results**: Adding the various concentrations, nBB showed potential inhibition during agonist-induced contractions (0.1–100 µM). Also, neurogenic contractions of rPx by EFS (2–32 Hz) were reduced by up to 57%. **Conclusions**: Overall, this study reports on an efficient protocol of nBB isolation from *A. smeathmannii* and its contractility effects on human prostate smooth muscle. Potentially, this could contribute to the natural production of BB from *A. smeathmannii* species while giving it evolutionary recognition. However, since BB influences prostate smooth muscle contractility, caution in patients taking herbal supplements containing nBB is essential, as this may play a role in contributing to the symptoms of urinary tract conditions.

## 1. Introduction

*Acridocarpus* Guill. & Perr. is one of the exceptional genera in the Paleotropics, as it has some floral morphology characteristics [1,2]. This genus comprises over thirty species of scandent and erect shrubs able to adapt to arid climates and is distributed in West Africa, East Africa, Madagascar, and New Caledonia [3]. The ethnobotanical use of *Acridocarpus smeathmannii* (DC.) Guill. & Perr (*A. smeathmannii*) was premised on its use for problems of the reproductive system and its accessory organs [4].

Our previous studies demonstrated the medicinal potential of crude *A. smeathmannii* root extract to improve reproductive behavior and functions in animal models [5]. Also, its toxicological assessment showed that the extract was relatively safe in rodents [6]. The phytochemical analysis of this extract revealed a mixture of polyphenols, flavonoids, sesquiterpene hydrocarbons, fatty acids, and benzyl alcohol esters [5]. To identify an active principle on a molecular basis, we further purified *A. smeathmannii* root extract by liquid–liquid extraction, column chromatography, and preparative thin-layer chromatography (TLC). Gas chromatography–mass spectrometry (GC-MS), high-performance liquid chromatography (HPLC-UV-VIS), and NMR spectroscopy led to the characterization of a natural benzyl benzoate (nBB). Its identity was finally confirmed by a comparison with the spectroscopic data of a commercial BB sample.

The chemical determination and physicochemical characteristics of BB have been reviewed [7,8,9]. BB is a popular old drug known for its spasmolytic effects [10] and vasodilation [11]. It is naturally found in various essential oils present in Peru and Tolu balsams, as well as being an abundant compound in several plant roots, flowers, and stems [12]. In the health industry, BB has been applied to smooth muscle tissue problems related to the genito-urinary apparatus, including the urinary bladder, ureter, and uterus [13]. Moreover, it was given to patients with dysmenorrhea before the discovery of other preferable analgesics, antipyretics, and anti-inflammatory agents [14]. It also relieves asthma and related symptoms [15]. Currently, it is applied to the skin as a lotion for ticks and lice [16] and is used as a preservative in heparin drugs [17], as well as a vehicle in testosterone preparations for treating hypogonadism [18]. Surprisingly, the mechanism of action of BB is still unknown. Recent studies have suggested that BB helps modulate the spastic effects of smooth muscle viscera in patients, thereby offering relief in acute spastic conditions [19].

The problems of lower urinary tract symptoms (LUTSs) are on the increase. The pathological and genetic bases of LUTS-associated risk factors have been updated [20,21,22]. Despite the many years of efforts towards a pharmacological intervention, a symptomatic LUT responds to α_1_-adrenergic receptor antagonists for flow rates of urine control, but this does not alleviate the long-term risk of urinary retention [23,24]. Also, 5α-reductase inhibitors, which reduce dihydrotestosterone production, can help correct volume-related symptoms and decrease the risk of acute urinary retention [25]. When combined, α_1_-adrenergic receptor antagonists and 5α-reductase inhibitors can achieve more therapeutic goals in LUTSs suggestive of benign prostate hypertrophy (BPH) [26]. Another relevant second line of treatment is less effective and is not preferred [22,27]. In addition to this, medications are complicated by adverse drug effects and patient intolerance, and the patient may still proceed to surgery. Importantly, there is no ideal drug for the treatment of mixed LUTSs [27]. These aspects necessitate a search for a new medical intervention or an alternative therapy. To stem the tide, the European Association of Urology guidelines have for the first time supported the recommendation of medicinal plant products as supplementary treatments for the alternative management of LUTSs [23]. Several herbal remedies or medicinal plant products have been reported for use in LUTSs suggestive of BPH [28,29]. There is growing interest in addressing the gaps between complex phytochemicals and drug discovery [30]. Interestingly, the natural compound production of bioactives could promote the commercialization of a specific plant or genus. Thousands of compounds in plant extracts with known ethnobotanical histories have been screened for biological activity [29,30,31]. Importantly, the purification of compounds from natural sources could harness an understanding of the evolutionary traits of a plant group and its importance. One main goal of this study is to potentially develop a bioactive source of nBB from the *A. smeathmannii* species while giving it evolutionary recognition. There is a paucity of studies on the in vitro effects of nBB action on human prostate smooth muscle tissue. The present study is the first to report on the isolation and purification of nBB from *A. smeathmannii*. Also, it is the first report on the effects of nBB from this genus on the activity of the human prostate smooth muscle.

## 2. Results and Discussion

### 2.1. Structural Analysis of HLASFF12

**Figure 1:**
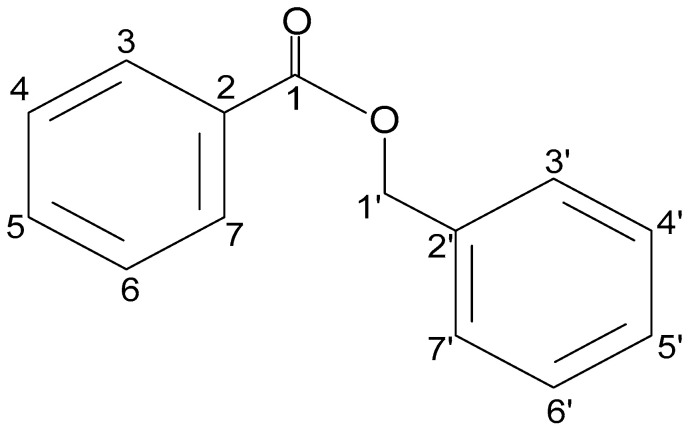
Structural elucidation of HLASFF12 (nBB).

*A. smeathmannii* root extract has been used for a long in Western Africa and has become a constituent of many polyherbal mixtures [4]. In retrospect, nBB is one of the most abundant components in *A. smeathmannii* root extracts. Thus, in pursuit of its phytoactive components, repeated-column chromatography led to the fraction HLASFF12, which we identified by GC-MS, HPLC, and NMR (1D and 2D) as a natural benzyl benzoate (see Supplementary Materials). Thus, HLASFF12 containing nBB as the major compound also constitutes one of the main compounds in *A. smeathmannii* root extracts [5]. This study provides the first report of nBB (HLASFF12) (Figure 1) isolated from *A. smeathmannii* roots and further explores its potential modulatory effects on human prostate smooth muscle tissue in an organ bath.

A reversed-phase HPLC analysis of the peak of fraction HLASFF12 had a retention volume of 22.4 mL. We performed a more detailed structural analysis by NMR (Supplementary Figures S3–S6). The GC analysis of HLASFF12 again showed a single peak at a retention time of 18.92 min (Supplementary Figure S1). In the mass spectrum, HLASFF12 was characterized by a molecular ion M+ at m/z 212 and a base peak at *m*/*z* 105 (Supplementary Figure S2). A similarity search with data from the NIST library resulted in a 98% match with the mass spectrum of benzoic acid benzyl ester (benzyl benzoate, nBB) (Supplementary Figure S2). The ^1^H NMR spectrum (500 MHz, CDCl_3_) was characterized by intense signals in the spectral region for aromatics (i.e., 8.2–7.3 ppm) (Supplementary Figure S3). Moreover, we observed a singlet peak at 5.42 ppm, which was determined as a CH_2_ group by a two-dimensional HSQC-DEPT experiment (Supplementary Figures S4–S6). Normalizing the integral of this signal to an arbitrary value of 2, the total integral of the signals in the aromatic region indicated the presence of 10 H-atoms, as expected for BB. Fully in line with the expected structure, NMR clearly showed the two benzyl moieties of nBB connected via a CH_2_ group that is attached to an O-atom. The presence of two benzyl rings was also confirmed by the observed correlation signals in the two-dimensional COSY, NOESY, HSQC, and HMBC spectra (Supplementary Figures S3–S6). Overall analyses of the ^1^H NMR spectrum of HLASFF12 did not display other signals at high intensities and the TLC, GC, and HPLC data only showed one signal for nBB (>90%) (Supplementary Figure S7).

### 2.2. Effects of HLASFF12 on Noradrenaline-Induced Contractions of Human Prostate Tissues

**Figure 2:**
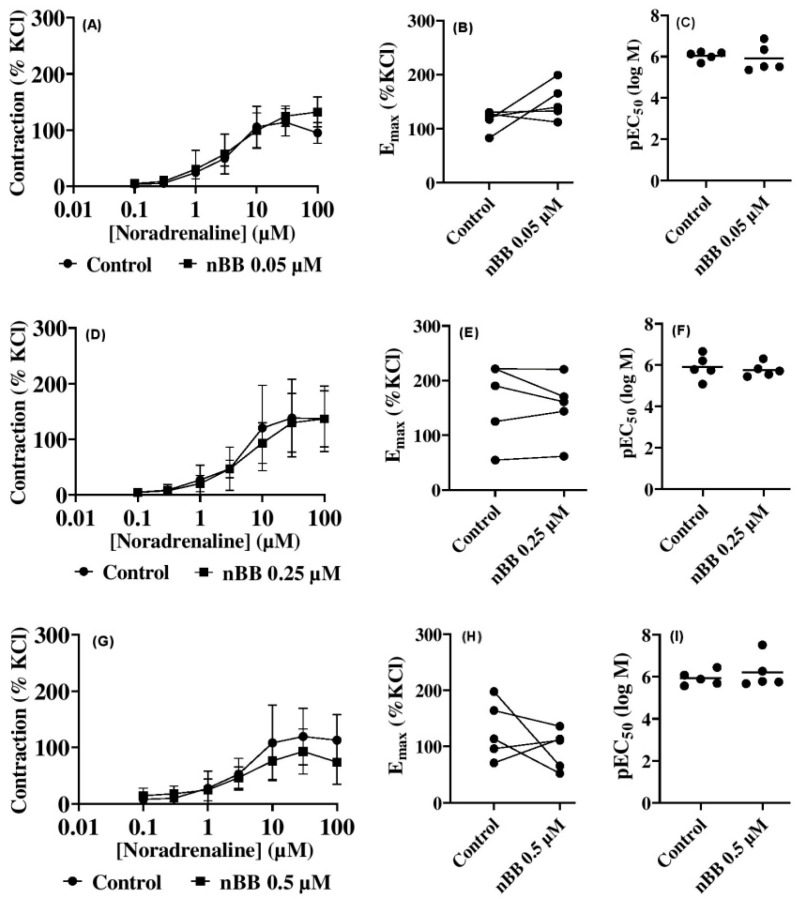
Effects of benzyl benzoate from *A. smeathmannii* extract on noradrenaline-induced contraction of human prostate smooth muscle tissue e.g., nBB at 0.05 µM (**A**), 0.25 µM (**D**) and 0.5 µM (**G**). The E_max_ effects of nBB at 0.05 µM (**B**), 0.25 µM (**E**) or 0.5 µM (**H**). The pEC_50_ effects of nBB for 0.05 µM (**C**), 0.25 µM (**F**) or 0.5 µM (**I**). Results are expressed as means ± SD (*n* = 5 patients per series, with tissue from each patient split into both the nBB and control group). nBB: natural benzyl benzoate. Tensions are expressed as % of high molar KCl-induced contraction assessed prior to the application of the control and nBB. E_max_ and pEC_50_ were calculated by curve fitting for each experiment.

In the current study, the addition of NA following incubation with nBB (0.05 µM, 0.25 µM, and 0.50 µM) in an organ bath was used to evaluate human prostate smooth muscle contractility against a control group (Figure 2A–I) (Table 1). Thus, 0.05 µM nBB added to the bath showed an increase in NA-induced contractility of the human prostate (*p* > 0.05) of up to 39% at 0.50 µM NA (mean difference (MD) 37.23 [−75.22–0.77] % of KCl). In contrast, inhibitions of NA-induced contractions of the human prostate were obtained with increasing nBB concentrations (0.25 and 0.50 µM). Thus, at 10, 30, and 100 µM NA, the addition of 0.25 µM produced inhibitions (*p* > 0.05) of up to 22% (MD 27.11 [−8.985–63.20] of % KCl), 6.2% (8.512 [−27.58–44.60]), and 0.1% (0.11 [−35.98–36.20]) when compared with the ethanol control group. Furthermore, at 3, 10, 30, and 100 µM NA, incubation with 0.5 µM nBB produced inhibitions (*p* > 0.05) of up to 11.8% (MD 6.19 [−62.22–74.60]), 29.6% (32.07 [−36.34–100.50 µM]), 22.1% (26.39 [−42.02–94.80]), and 34.6% (39.04 [−29.37–107.5]). Our results show that nBB could influence the adrenergic response to human prostate smooth muscle contractility, at least to an extent. Whether this would translate into a clinical implication or not is very hard to tell. Although there are divergent opinions about their safety in the reproductive system, nBB and benzyl derivatives remain relevant. However, this calls for a further evaluation of their biological effects, in particular given the fact that they are important for clinical and non-clinical uses.

Several medicinal plants which are easily accessible, environmentally friendly, and cost-effective—especially for those from low-income and developing countries—are being explored for the production of nBB and other benzyl derivatives [10,32]. Even though nBB has been previously synthesized and is available in synthetic form, our study is the first to report on nBB isolation from *A. smeathmannii*. These findings contribute to the phytochemical profiling of the plant and highlight the relevance of natural sources for drug discovery. Few studies have reported on the isolation and purification of benzyl benzoates from medicinal plants [33,34,35,36] and explored their potential relaxation and antibacterial uses. Recently, there have been reports that benzyl radicals exhibit smooth muscle-relaxing actions with minimal toxicity [37]. nBB, an ester soluble in many organic compounds such as alcohol, but less so in water, is a well-known cosmetic ingredient and pesticide [7,35]. Now, nBB is chemically synthesized from the condensation of benzoic acid and benzyl alcohol, among others [8]. nBB is easily metabolized and has a multipurpose use [35]. The exposure, uses, and controversy of benzoic acid and its derivatives in several foods and additives have been reviewed [38,39,40]. Human clinical studies indicate that the pharmacokinetics of benzoic acid and its salts are similar in children and adults, and they possess minimal toxicity, which may increase under chronic conditions [41,42]. The safety assessment of nBB, benzyl alcohol, benzoic acid, and its salts has been documented [35,38,39].

### 2.3. Effects of HLASFF12 on Phenylephrine-Induced Contractions of Human Prostate Tissues

**Figure 3:**
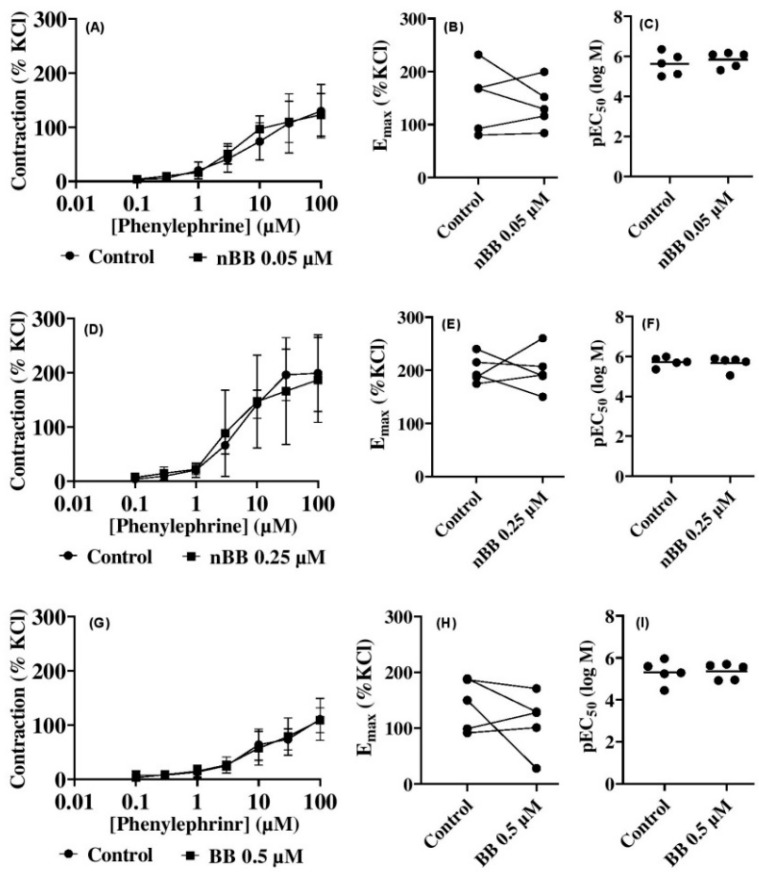
Effects of benzyl benzoate from *A. smeathmannii* extract on phenylephrine-induced contraction of human prostate smooth muscle tissue e.g. nBB at 0.05 µM (**A**), 0.25 µM (**D**) and 0.5 µM (**G**). The E_max_ effects of nBB at 0.05 µM (**B**), 0.25 µM (**E**) or 0.5 µM (**H**). The pEC_50_ effects of nBB for 0.05 µM (**C**), 0.25 µM (**F**) or 0.5 µM (**I**). Results are expressed as means ± SD (*n* = 5 patients per series, with tissue from each patient split into both the nBB and control group). nBB: natural benzyl benzoate. Tensions are expressed as % of high molar KCl-induced contraction assessed prior to athe pplication of the control and nBB. E_max_ and pEC_50_ were calculated by curve fitting for each experiment.

Here, the contraction of human prostate tissues was achieved by phenylephrine (PHE), an α_1_-adrenoceptor agonist (0.1–100 µM PHE) in (Figure 3A–I) (Table 1). Thus, the lowest nBB dose (0.05 µM) lowered (*p* > 0.05) the peak by 5% at a 0.5 µM PHE concentration (MD 6.7 [−43.65–57.12] % of KCl). Also, slight but insignificant inhibitions of PHE-induced contractions were observed with an increasing nBB (*p* > 0.05) concentration of 0.25 µM nBB, with a peak at 30 µM PHE of up to 9.2% (MD 29.97 [−53.58–113.5] of % KCl) and a further decrease (*p* > 0.05) at 100 µM to 2% (12.31 [−71.24–95.86] of % KCl) when compared with the ethanol control group. However, at 0.5 µM nBB, a reduction of 1.6% (MD 1.75 [−26.77–30.27] of KCl) in PHE-induced contraction was observed at 100 µM PHE. Both males and females can be affected by LUTSs, which are on the increase [12,43]. Symptomatic LUTSs are common in men, particularly because of the prostate enlargement secondary to BPH [43,44]. BPH mostly affects men in their fifties and above, and it may impact their overall health [22]. Given the pathophysiology of BPH, increased prostate smooth muscle tone is a risk factor for urethral obstruction, causing impairments that precipitate LUTSs [44]. The α_1_-adrenoceptor remains the most sought after in the management of LUTSs. Our results showed that the doses of nBB applied did not significantly alter the contractility of human prostate smooth muscle, although the highest dose slightly lowered the E_max_ (Figure 3H).

### 2.4. Effects of HLASFF12 on Electric Field Stimulation-Induced Contraction of Human Prostate Tissues

**Figure 4:**
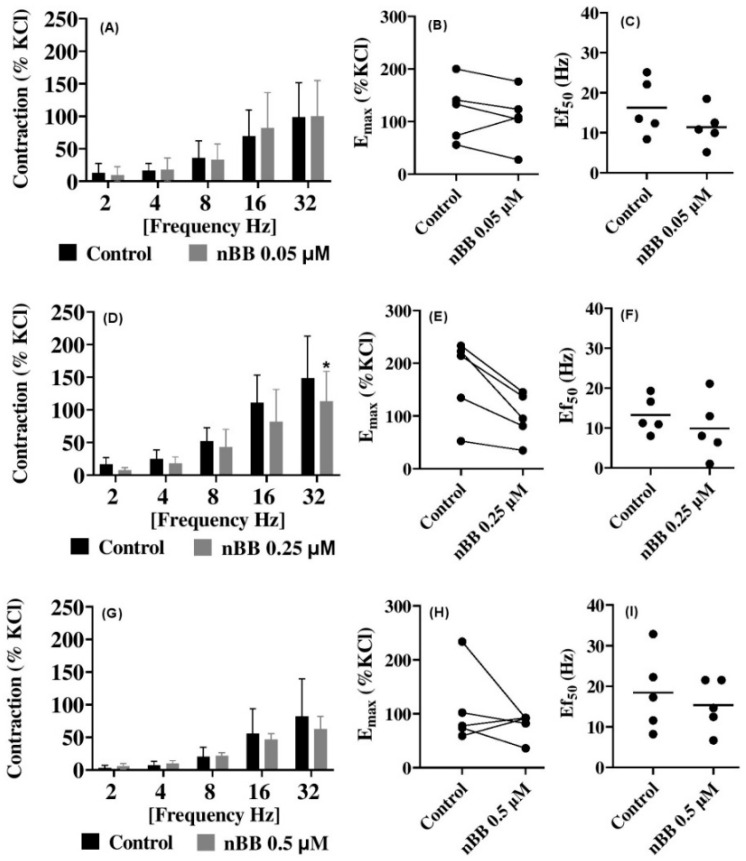
Effects of benzyl benzoate from *A. smeathmannii* extract on EFS-induced prostate smooth muscle contraction e.g. nBB at 0.05 µM (**A**), 0.25 µM (at 32 Hz, * *p* < 0.01) (**D**) and 0.5 µM (**G**). The E_max_ effects of nBB at 0.05 µM (**B**), 0.25 µM (**E**) or 0.5 µM (**H**). The Ef_50_ effects of nBB for 0.05 µM (**C**), 0.25 µM (**F**) or 0.5 µM (**I**). Results are expressed as means ± SD (*n* = 5 patients per series, with tissue from each patient split into both the nBB and control group). Tensions are expressed as % of high molar KCl-induced contraction assessed prior to the application of the control ethanol and nBB. E_max_ and Ef_50_ were calculated by curve fitting for each experiment.

The release of endogenous neurotransmitters producing neurogenic potentials by EFS, leading to contraction, was investigated in human prostate tissues (Figure 4A–I) (Table 2). The addition of nBB produced no inhibition during EFS-induced contractions induced by 2–32 Hz in human prostate tissues. All the frequencies applied (2–32 Hz) at 10 µM nBB did not produce any change but further increased contractions of human prostate tissues when compared with the control. However, 50 µM nBB reduced (*p* > 0.05) EFS-induced human prostate contractions within the curve, with a peak of up to 57% at 2 Hz (MD 10.51 µM [−15.58 to 36.61] of KCl), 4 Hz (27.4%) (6.8 [−19.32 to 32.87]), 8 Hz (17%) (12.68 [−13.44 to 38.75]), 16 Hz (26.8%) (23.7 (−2.351 to 49.84] of KCl, *p* = 0.08), and 32 Hz (22%) (41.31 [15.22 to 67.41] of KCl, *p* = 0.01). With 0.5 µM nBB, inhibitions were seen (*p* > 0.05), with a peak at 16 Hz (16%) (9.04 [−36.45 to 54.53] and 32 Hz (23.8%) (19.52 [−25.97 to 65.01] of KCl) when compared with the control groups.

These findings show that persistent firing waves discharged from the prostate and the increased tone following the application of low-dose nBB may increase prostatic urethral pressure further, which could worsen patient symptoms. Thus, the incubation of nBB in the bath to human prostate smooth muscle contractility offered some degree of inhibition, although this was insignificant, giving further credence to its anti-spasmolytic activity [10,32].

Based on our results, it may be that nBB works together with α_1_-adrenergic to promote prostate stimulation. Studies have suggested that non-adrenergic contraction efforts, including those of endothelins, contribute to these efforts [21], or that inhibitory action favors non-selective adrenergic inhibition and possibly other neurotransmitter release networks. However, the difference between nBB-treated samples and those with the control vehicle was large enough to reduce abnormal prostate electrical field stimulation at increasing dosages. Whether this would translate into clinical implication requires further investigation. The molecular mechanism of nBB in inhibiting prostate smooth muscle has not been studied, but these findings may point to the mechanism of vasodilation reported earlier [10,11,15]. This may also explain nBB’s inhibitory actions, associated with the depolarization of the nervous system. It positions us to explore whether nBB may directly interact with α-adrenergic receptors, modulating calcium channel activity or intracellular calcium release, since the excitation of smooth muscle depends on the same in most cases; the modulation of neurogenic contractions to achieve an influence on neurotransmitter release and/or post-synaptic receptor sensitivity are possible fields of study that are essential in terms of their health implications. The authors acknowledge their limited access to any patient data, and this study focused on experimental investigations rather than on the pharmacological management of patients, since we did not recruit patients directly. Thus, we applied a single tissue, split between the nBB and control groups, per investigation. It is assumed that whatever impact this may have on contractility should be distributed across groups. Given this, future studies will further explore in vivo studies and/or clinical trials to assess the safety and efficacy of nBB-containing formulations.

## 3. Materials and Methods

### 3.1. Drugs and Chemicals

Phenylephrine and noradrenaline were obtained from Sigma (Munich, Germany). Carbachol, methacholine, *n*-heptane (EMPLURA^®^), and deuterated chloroform (CDCl_3_) were purchased from Sigma-Aldrich (Munich, Germany), and potassium chloride was acquired from Sigma-Aldrich (St. Louis, MO, USA). Commercial benzyl benzoate (BB) was purchased from Sigma-Aldrich, Cheme GmbH, Schnelldorf, Germany (PCode: 102808073). Other solvents and reagents used were of analytical grade.

### 3.2. Plant Collection, Authentication and Extraction

**Figure 5:**
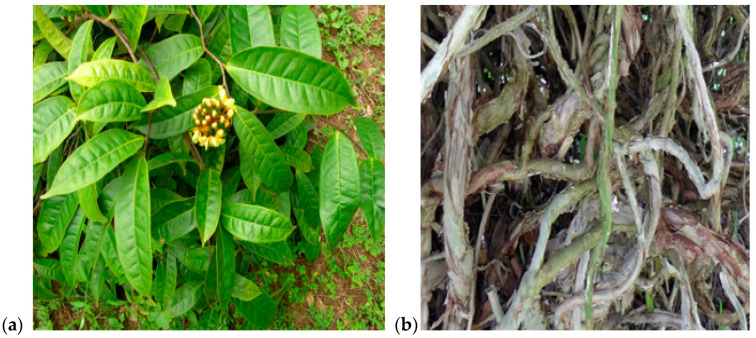
A picture of *Acridocarpus smeathannii* (DC.) Guill. & Perr (**a**) plant and (**b**) roots in its native environment (voucher specimen, FHI: 113685).

*A. smeathmannii* (DC.) Guill. & Perr roots were collected from natural farmland beside the premiere area, Ibadan, Oyo State, Nigeria, in 2023 by the botanist Dr. A. Samuel Odewo [Forest Research Institute of Nigeria (FRIN), Ibadan, Oyo-State, Nigeria]. Plant-authenticated vouchers were deposited in a publicly available herbarium, FRIN, Nigeria (voucher number, FHI: 113685) (Figure 5). Research permission to perform experiments with *A. smeathmannii* roots was approved by the Health Research and Ethics Committee, College of Medicine, University of Lagos (CMUL/HREC/09/18/424). In addition, the authors obtained a phytosanitary certification (No. 0124876) from the Nigeria Agricultural Quarantine Service Plant Health, Nigeria. The plant roots were dried at ±23 °C, expunged from the sticks, and ground into powder (Christy and Norris LAB MILL, NO. 50158, Suffolk, UK) in the Pharmacognosy Department, Olabisi Onabanjo University Nigeria.

A Soxhlet apparatus using *n*-heptane as a solvent was used for the extraction of *A. smeathmannii* roots. A solvent extractor thimble was filled with (25 g × 10) of *A. smeathmannii* root powder and extracted with *n*-heptane (250 mL flask) for four hours. The solvent was removed using a rotatory evaporator coupled with Vacuumbrand CVC 3000 and IKA^®^ RV 10 digital (IKA^®^, RV 10D S93, Baden-Württemberg, Germany) at 40 ± 1 °C and 120 mbar. The dried light golden-yellow extract (27.1 g; yield, 4.96%) was reconstituted and used in fractions (stored at 4 °C) for further purification and biological activity studies in vitro.

### 3.3. Chromatography Studies

Preparative TLC analyses were carried out on precoated silica gel 60 F254 and cellulose sheets (Merck, Darmstadt, Germany), and visualization of the plates was carried out under UV (Jeulin^®^, Jacques-Monod, Evreux Cedex, France, 365 nm) or via staining using potassium permanganate (0.1 M). The total extract of *A. smeathmannii* (AS) (27.1 g) was dissolved in 90% methanol and extracted in *n*-hexane (1:10) by a separating funnel into an upper layer (HUASF, 1.25 g) and a lower layer (HLASF, 15.24 g). Further, HLASF was dissolved in 70% methanol and extracted with dichloromethane to afford a dichloromethane fraction (HLASF) (0.91 g) and a residue (11.14 g), which were stored at −4 °C. For preparative purposes, the HLASF fraction was separated using an 18 cm column (diameter, 5 cm) filled with 30 g silica gel 60 (35–75 μm mesh, CAS No. 7631-89-9, Merck KGaA). The column was sealed and topped with sea sand (3 mm, Cat. Nr. 1313710000, Grussing GmbH, Filsum, Lower-Saxony, Germany). The flow rate was 2.5 mL/min. Using a 5:1:0.02 mixture of *n*-hexane/ethyl acetate/acetic acid (*v*/*v*), 44 fractions (5 mL each) were collected. HLASF fractions 1–44 were monitored by preparative TLC using a mixture of *n*-hexane/ethyl acetate/acetic acid (10:1:0.02, *v*/*v*). Fractions showing spots with the same R_f_ value were gathered in a single flask, and the solvent was removed using a rotary evaporator. Repeated-column chromatography of HLASF 7–14 yielded 22 fractions (HLASFF 1–22). Serial TLC, NMR, HPLC, and GC-MS were used to analyze the isolates. HLASFF12 (nBB) appeared as a colorless liquid that crystalized into a white solid (10.5 mg). Retention on TLC plates was measured as the retention factor (Rf). The preparative TLC technique afforded a R_f_ value of 0.53 for HLASFF12 from HLASF.

### 3.4. Gas Chromatography–Mass Spectrometry Analysis

Gas chromatography–mass spectrometry (GC-MS) analysis was performed with a QP2010 Plus gas chromatograph/mass instrument (Shimadzu^®^, Kyoto, Japan) equipped with a fused silica capillary column (Equity TM-5; 30 m, 0.25 mm, 0.25 µm film thickness; SUPELCO, Bellefonte, PA, USA) and a quadrupole detector working with electron impact ionization at 70 eV. An aliquot (30 µg in 500 µL methanol) of *A. smeathmannii* was prepared. A fraction of 1 µL was injected in 1:5 split modes at an interface temperature (50 °C for 3 min and 50–310 °C with 10 °C/min) and a helium inlet pressure of 70 kPa. Shimadzu^®^ (Kyoto, Japan) GC-MS scan measurement was used for data collection in duplicate.

### 3.5. Nuclear Magnetic Resonance Analysis

Nuclear magnetic resonance (NMR) spectroscopy was performed with 5 mg of *A. smeathmannii* dissolved in 500 μL CDCl_3_. ^1^H-NMR spectra were registered at 27 °C with an Avance-III 500 MHz system (BRUKER, Ettlingen, Germany) equipped within an inverse probe head (5 mm SEI, ^1^H/^13^C; Z-gradient). Two-dimensional experiments including COSY, HMBC, HSQC, and NOESY spectra were measured using standard Bruker parameter sets in TOPSPIN 3.5 software. Data processing and analysis were performed with MNova (15.0.1).

### 3.6. High-Performance Column Chromatography (HPLC)

High-Performance Column Chromatography (HPLC) (Shimadzu^®^ SIL-20AHT Europa GmbH, Duisburg, Germany) was carried out with HPLC instruments (SPD-10AV UV-VIS detector, LC-10AS, SIL-20A HT autosampler) using a LiChrospher^®^ column (RP-18, 5 µm). Thus, HLASF1-44 and HLASFF12 (5 µL each) aliquots were dissolved in 1 mL methanol and placed in glass vials, after which they were filled up to near the total vial volume of 1.5 mL and placed in the HPLC (Shimadzu^®^) sampler. The column was developed with aqueous methanol (60%) at 0.5 mL/min. The eluate was monitored at 280 nm.

### 3.7. Biological Actions of HLASFF12 on Human Prostate Tissue Contraction

This study obtained tissues from the periurethral zone of the prostate from patients who underwent radical prostatectomy (rPx) for prostate cancer. Tissues were sampled immediately after surgery, followed by macroscopic examination of the tissues by the pathology department (LMU). Specifically, periurethral zone portions were taken and used, since most prostate cancers arise in the peripheral zone, so patients with previous transurethral resection or with previous laser enucleation were excluded. The specimens were opened by a single longitudinal cut from the capsule to the urethra for macroscopic inspection and sampling. Both surfaces were macroscopically examined for any obvious tumor infiltration. Further, experimental tissues were prepared from the transitional periurethral zone. Thus, prostates with macroscopically visible tumors in the periurethral zone were excluded from sampling. This was rare (<1% of prostates), as most prostate tumors are located in the peripheral zone. However, it is noteworthy that radical prostatectomy tissues are not specifically representative of BPH and do not cover medication-refractory voiding symptoms in BPH [45]. For storage and transport, organs and tissues were placed in a Custodiol^®^ (Köhler, Bensheim, Germany) solution. Most importantly, BPH is present in almost 80% of patients with prostate cancer. For the measurement of contraction, dissected prostate strips (6 × 3 × 3 mm) were mounted in 10 mL aerated (95% O_2_ and 5% CO_2_) tissue baths (Danish Myotechnology, Hinnerup, Denmark) with four chambers, containing Krebs–Henseleit (KH) solution (37 °C, pH 7.4) [26]. Experiments were started within 1 h of tissue collection sampling. Mounted preparations were stretched to approximately 4.9 mN and left to equilibrate for 45 min. The initial phase of the equilibration period may be characterized by spontaneous decreases in tone; therefore, tension was adjusted three times during the equilibration period until a stable resting tone of 4.9 mN was attained. After the equilibration period, the maximum contraction induced by 80 mM KCl was assessed. Subsequently, media were rinsed out three times with KH solution, and nBB or ethanol (for controls) was added. Cumulative concentration–response curves for noradrenaline (NA) and phenylephrine (PHE) (for prostate tissues) were constructed 30 min following the addition of nBB or ethanol. Each patient’s sample was split into a control and nBB group (two specimens in each) and was examined and analyzed as a single case within the same experiment. Different patient samples were used for agonist-induced contraction. Consequently, both groups in each series had identical group sizes. Agonist-induced contractions were expressed as a percentage of 80 mM KCl-induced contractions (maximum of phasic contraction) [26]. This is required because of differences in stromal/epithelial ratios, different smooth muscle content, varying degrees of BPH, and/or any other heterogeneity between tissue samples and patients. A total of 50 µL nBB was added to a 10 mL organ bath to achieve final bath concentrations of 0.05, 0.25, and 0.5 µM nBB, and this was compared with the control (ethanol) group. The maximum possible contractions (E_max_), concentrations inducing 50% of maximum agonist-induced contraction (EC_50_), and frequencies (f) as 50% of maximum EFS-induced contraction (Ef_50_) were calculated separately for each single experiment by curve fitting using GraphPad Prism (GraphPad Software Inc., San Diego, CA, USA). EC_50_ values were expressed as negative logarithms of the molar concentration for agonists (pEC_50_) to quantify potency [26]. Studies on human prostate tissues were carried out in line with the Declaration of Helsinki of the World Medical Association and were approved by the ethics committee of Ludwig-Maximilians University (LMU), Munich, Germany (LMU/MH060922). LMU Klinikum, Munich, Germany, was responsible for informed consent, which was obtained from all patients.

### 3.8. Effects of HLAFF12 on Prostate Smooth Muscle Contractile Activity

The possible modulatory effects of increasing nBB concentrations on prostate tissues were assessed and compared with the controls. Contractions were induced with NA (non-selective) and PHE (α_1_-selective) agonists for adrenoceptors (0.1–100 µM) in prostate tissues. Anti-contractile effects were expressed as a positive percentage in proportion to the contraction achieved by an agonist.

### 3.9. Electrical Field Stimulation

Thirty minutes after the addition of nBB or the control (ethanol), electrical field stimulation (EFS) was applied to achieve frequency–response curves for contractions induced by neurogenic activation. EFS generates action potentials that cause endogenous neurotransmitters, including noradrenaline and acetylcholine, to be released. Briefly, tissue strips were placed between two parallel platinum electrodes connected to a CS4 stimulator (Danish Myotechnology). Square pulses (positive monopole) with a duration of 1 millisecond and a voltage of 20 V were applied for the duration of the training. EFS-induced contractile responses were studied at frequencies of 2, 4, 8, 16, and 32 Hz, with train intervals of 60 s between stimulations. Similarly, only one curve was recorded with each sample. Calculations of the EFS-induced contractions followed the measurement of peak height in EFS-induced contractions expressed as % of 80 mM KCl-induced contractions (maximum of phasic contraction). The Emax values and frequencies (f) inducing g 50% of the maximum EFS-induced contraction (Ef_50_) were evaluated by curve fitting using GraphPad Prism.

### 3.10. Data and Statistical Analyses

The results of concentration–response and frequency–response curves are presented as means ± standard deviation (SD). Post hoc analyses for multiple comparisons at single-agonist concentrations or frequencies among groups were compared by a two-way ANOVA for multiple comparisons. Each series of organ bath experiments is based on *n* = 5 independent experiments, including values from paired samples in each experiment. Statistical analyses were performed using GraphPad Prism (9.5.0) (GraphPad Software Inc., San Diego, CA, USA). Emax, pEC_50_, and *Ef*_50_ values were means of two samples in each series in the human prostate experiments compared by a paired *t*-test [27]. The changes in concentration and frequency responses observed in contraction experiments are reported as percentage change relative to the control (mean difference (MD) with 95% CIs of KCl).

## 4. Conclusions

This study demonstrates the first-time isolation and purification of nBB from *A. smeathmannii* and its first recognition for this species. Also, the compound exhibits potential modulatory and inhibitory effects on adrenergic agonists and on the electrical field stimulation of human prostate smooth tissue. This is in support of the ethnobotanical usage of the plant’s crude extracts in the management of reproductive diseases. Also, since nBB modulates prostate smooth muscle contractility, caution is essential in patients taking herbal supplements or pharmaceutical formulations containing nBB, as this may play a role in contributing to the symptoms of urinary tract conditions.

## Figures and Tables

**Table 1:** Mean differences (MDs) for agonist-induced contractions after application of HLASFF12 (nBB) or control and 95% confidence intervals (CIs) (in parentheses, low to high) (% of KCl-induced contractions).

				Agonist Concentrations					
			0.1 μM	0.3 μM	1 μM	3 μM	10 μM	30 μM	100 μM
Prostate	nBB 0.05	Noradrenaline	−1.3 [−39.3 to 37.6]	−3.3 [ −41.3 to 34.7]	−6.2 [ −44.2 to 31.8]	−7.8 [ −45.8 to 30.2]	6.8 [ −31.2 to 44.8]	−11.1 [ −49.1 to 26.9]	−37.2 [ −75.2 to 0.77]
	nBB 0.25		0.4 [ −35.8 to 36.4]	1.1 [ −35.0 to 37.2]	6.5 [ −29.6 to 42.6]	0.5 [ −35.6 to 36.6]	27.1 [ −9.0 to 63.2]	8.5 [ −27.6 to 44.6]	0.1 [ −36.0 to 36.2]
	nBB 0.5		−6.6 [ −75.1 to 61.8]	−8.1 [ −76.6 to 60.3]	2.9 [ −65.6 to 71.3]	6.2 [ −62.2 to 74.6]	32.1 [ −36.3 to 100.5]	26.4 [ −42.0 to 94.8]	39.0 [ −29.4 to 107.5]
			0.1 Μm	0.3 μM	1 μM	3 μM	10 μM	30 μM	100 μM
Prostate	nBB 0.05	Phenylephrine	−0.3 [ −50.6 to 50.1]	−4.2 [ −54.6 to 46.2]	3.7 [ −46.7 to 54.1]	−9.2 [ −60.1 to 40.7]	−23.1 [ −73.5 to 27.3]	−2.7 [ −53.1 to 47.7]	6.7 [ −43.7 to 57.1]
	nBB 0.25		−2.8 [ −86.3 to 80.8]	−6.4 [ −89.0 to 78.1]	−1.4 [ −85.0 to 82.1]	−22.6 [ −106.2 to 60.9]	−5.0 [ −88.5 to 78.6]	30.0 [ −53.6 to 113.5]	12.3 [ −71.2 to 95.9]
	nBB 0.5		4.5 [ −24.0 to 33.0]	−0.9 [ −29.5 to 27.6]	−1.7 [ −30.3 to 26.8]	−1.6 [ −30.1 to 27.0]	−6.5 [ −22.0 to 35.0]	−5.2 [ −33.7 to 23.3]	1.8 [ −26.8 to 30.3]

Calculations were performed for those agonists and tissues where a possible inhibition of contraction by nBB was observed in concentration–response curves. nBB (0.05 µM), nBB (0.25 µM), and nBB (0.5 µM) are concentrations of the nBB compound. For each single experiment, contractions with the inhibitor were calculated as a percentage of the corresponding control in the same experiment and subtracted from the control (100 − (contraction with inhibitor)/(contraction control) × 100), i.e., between the inhibitor and the ethanol control group, for corresponding paired samples from the same prostate in each single experiment) and are expressed as MD with 95% CI. Results are expressed as means ± SD (*n* = 5 patients per series, with tissue from each patient split into both the nBB and ethanol control group).

**Table 2:** Mean differences (MDs) in EFS-induced contractions of the prostate, bladder, and porcine arteries after the application of nBB or control and 95% confidence intervals (CIs) (in parentheses, low to high) (% of KCl-induced contractions).

			Neurogenic Stimulations				
**Prostate**	**Freq.**		**2 Hz**	**4 Hz**	**8 Hz**	**16 Hz**	**32 Hz**
	nBB 0.05	EFS	3.7 [ −17.8 to 25.1]	−1.4 [ −22.9 to 20.1]	2.9 [ −18.6 to 24.4]	−12.2 [ −33.7 to 9.3]	−1.1 [ −22.6 to 20.4]
	nBB 0.25		10.5 [ −15.6 to 36.6]	6.8 [ −19.3 to 32.9]	12.7 [ −13.4 to 38.8]	23.7 [ −2.35 to 49.8]	41.3 [ 15.2 to 67.4]
	nBB 0.5		−2.6 [ −48.1 to 42.9]	−2.8 [ −48.3 to 42.7]	−1.5 [ −46.9 to 44.0]	9.0 [ −36.5 to 54.5]	19.5 [ −25.9 to 65.0]

Calculations were performed for those agonists and tissues where a possible inhibition of contraction by nBB was observed in concentration–response curves. nBB (0.05 µM), nBB (0.25 µM), and nBB (0.5 µM) are concentrations of the nBB compound. For each single experiment, contractions with the inhibitor were calculated as a percentage of the corresponding control in the same experiment and subtracted from the control (100 − (contraction with inhibitor)/(contraction control) × 100), i.e., between the inhibitor and the ethanol control group for corresponding paired samples from the same prostate in each single experiment) and are expressed as MD with 95% CIs. Results are expressed as means ± SD (*n* = 5 patients per series, with tissue from each patient split into both the nBB and control group).

## Data Availability

Data are contained within the article.

## Supplementary Materials

The following supporting information can be downloaded at https://www.mdpi.com/article/10.3390/ph18050687/s1: Figure S1: GC-MS chromatograms Analysis of *A. smeathmannii* extracts (a) HLSFF12 (b) HLSF 11–14 (c) HLSF 1–44 and (d) *A. Smeathmannii* root extract. Figure S2. GC-MS analysis of (a) HLASFF12 fraction (pink) and (b) standard reference (black) (c) benzyl benzoate; benzyl alcohol benzoic ester (m/z+ 212) (b) comparison using Labsolutions Shimazu^®^ by NIST. Figure S3. Nuclear Magnetic Resonance spectrum of (a) stacked ^1^H NMR HLASFF12 (a natural BB) vs. standard reference (BB) (2 vs. 1) (b) Integrated ^1^H NMR of HLASFF12. Figure S4: ^1^H-^1^H COSY spectrum of benzyl benzoate from *A. smeathamannii*. Figure S5: ^1^H-^13^C HSQC spectrum of benzyl benzoate from *A. smeathamannii.* Figure S6: ^1^H-^13^C HMBC spectrum of benzyl benzoate from *A. smeathamannii*. Figure S7 Spectrometry analysis of *A. smeathmannii* root extract fractions HPLC chromatograms (Aqueous MeOH, 60:40, 280 nm) (a) HLASF 1–44 and (b) HLASFF 12. Table S1: NMR Characteristics of HLASFF12.

## Abbreviations

*A. smeathmannii* (*Acridocarpus smeathmannii*)	(AS)
Benign prostatic hyperplasia	(BPH)
Benzyl benzoate	(BB)
Concentrations inducing 50% of maximum agonist-induced contraction	(EC_50_)
Electrical field stimulation	(EFS)
Frequencies (f) inducing 50% of maximum EFS-induced contraction	(Ef_50_)
Gas chromatography–mass spectrometry	(GC-MS)
High-performance liquid chromatography	(HPLC)
Lower layer of *A. smeathmannii*	(HLASF)
Lower layer of *A. smeathmannii* fraction	(HLASFF12) (nBB)
Lower urinary tract symptoms	(LUTSs)
Maximum possible contractions	(E_max_)
Negative logarithms of the molar concentration for agonists	(pEC_50_)
Noradrenaline	(NA)
Nuclear magnetic resonance	(NMR)
Phenylephrine	(PHE)
Radical prostatectomy	(rPx)
Retention factor	(R_f_)
Thin layer chromatography	(TLC)
Homonuclear correlation spectroscopy	(COSY)
Nuclear Overhauser Effect Spectroscopy	(NOESY)
Heteronuclear single quantum coherence	(HSQC)
Distortionless Enhancement by Polarization Transfer	(DEPT)
Heteronuclear Multiple Bond Correlation	(HMBC)
