# Supplementary material for: Benzyl Benzoate Isolation from Acridocarpus smeathmannii (DC.) Guill. & Perr Roots and Its Bioactivity on Human Prostate Smooth Muscle Contractions
Source: Pharmaceuticals (Basel). 2025 May 6;18(5):687. doi: 10.3390/ph18050687 (PMC12114539; doi:10.3390/ph18050687)
Supplement: Supplementary file 1 [file pharmaceuticals-18-00687-s001.zip › pharmaceuticals-3519315-supplementary.pdf]

Supplementary Figures

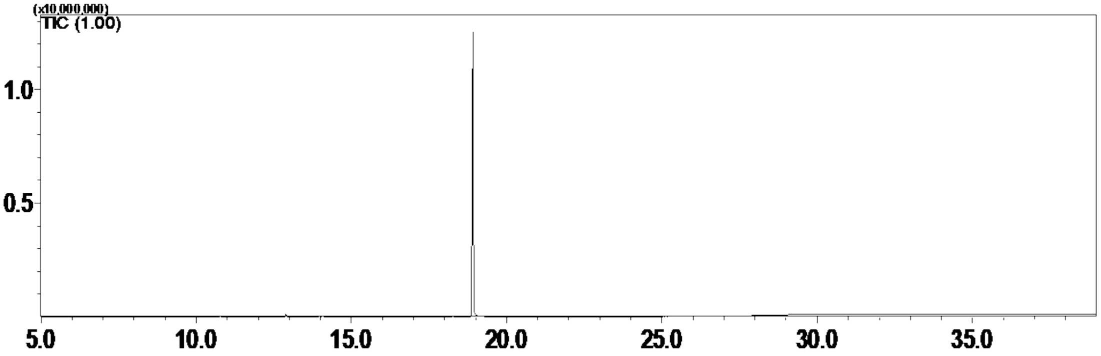

(a)

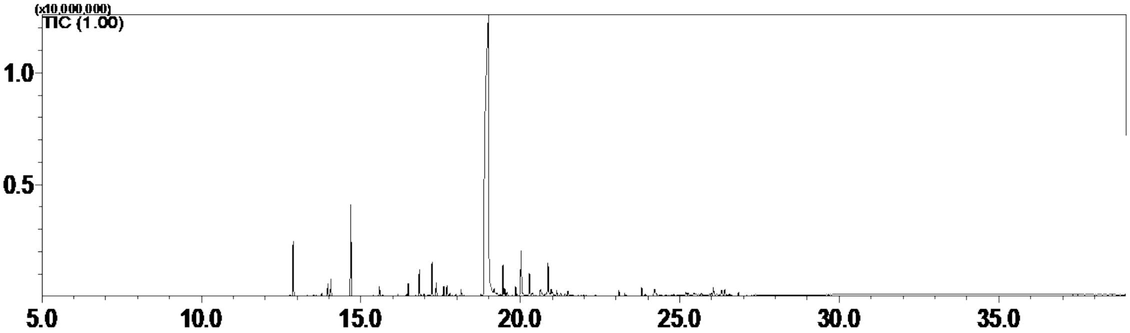

()

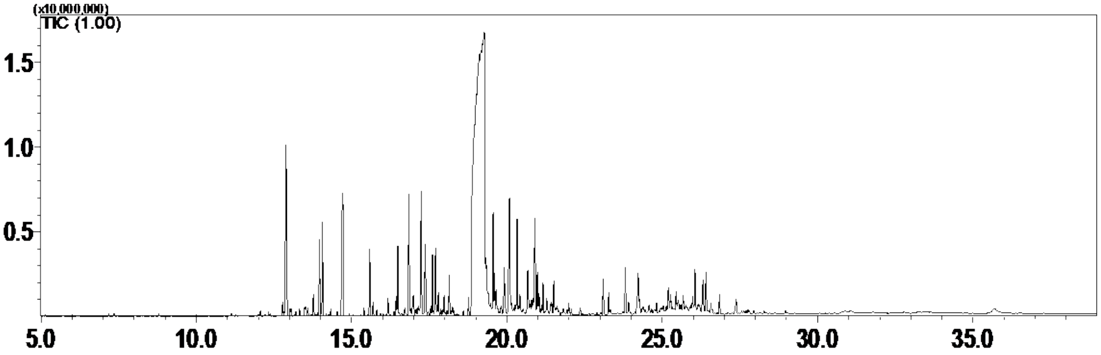

(c)

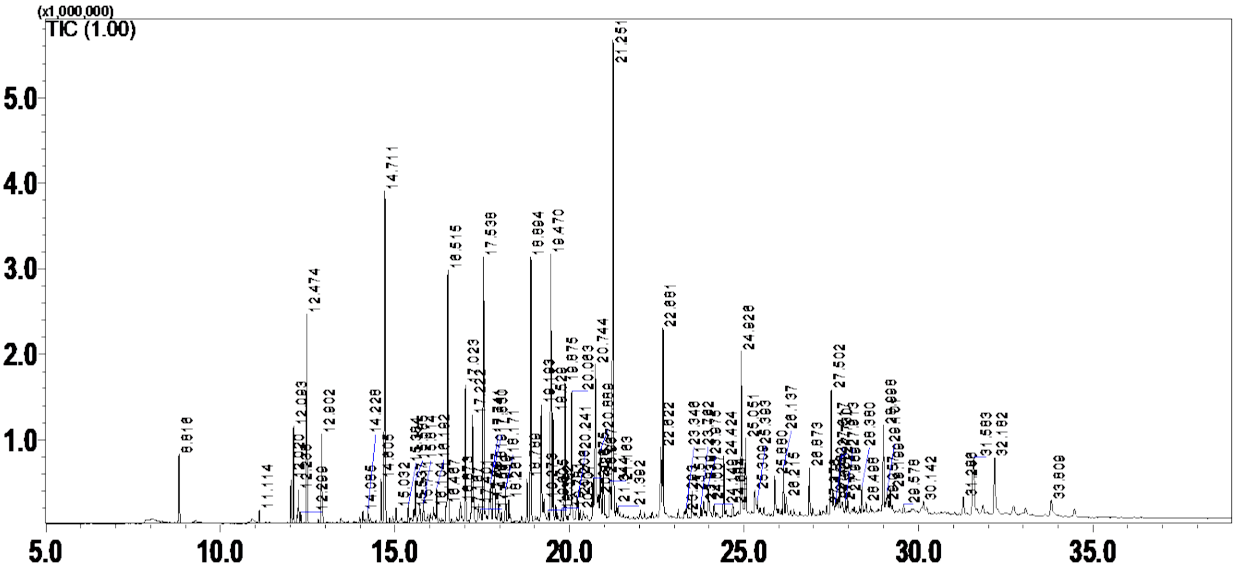

(d)

Figure S1: GC-MS chromatograms Analysis of *A. smeathmannii* extracts (a) HLSFF12 (b) HLSF 11 - 14 (c) HLSF 1 - 44 and (d) *A. Smeathmannii* root extract.

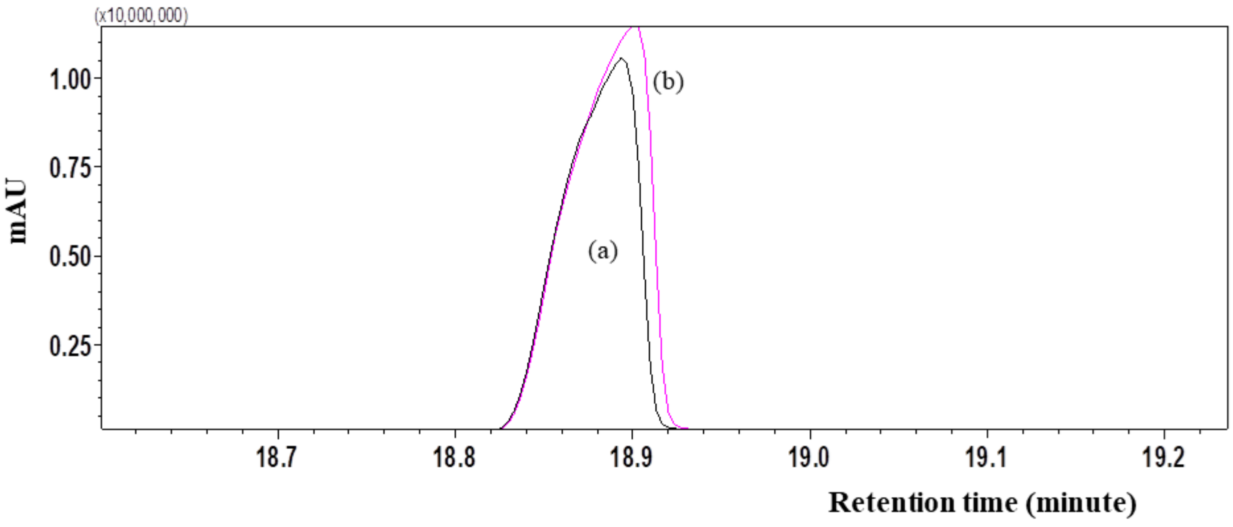

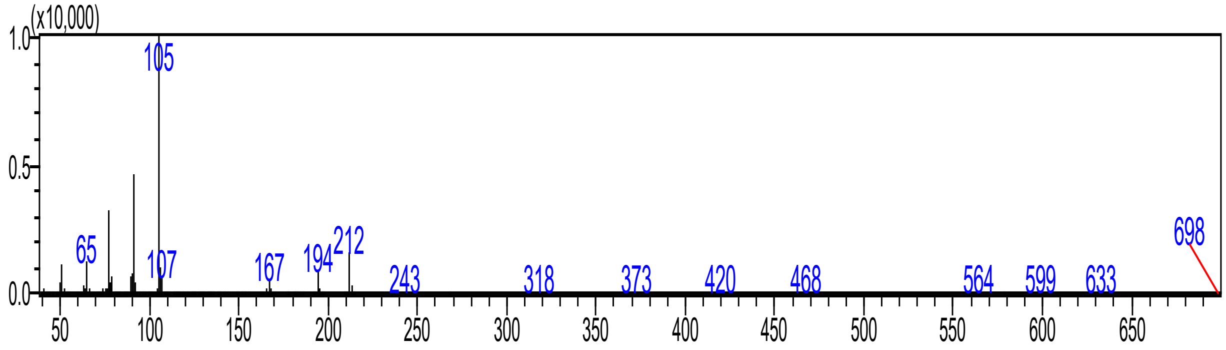

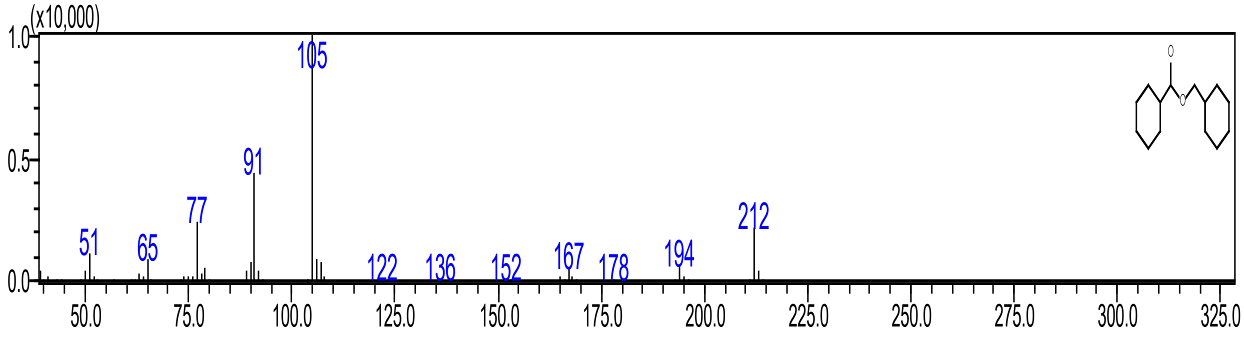

(c)

Figure S2. GC-MS analysis of (a) HLASFF12 fraction (pink) and (b) standard reference (black) (c) benzyl benzoate; benzyl alcohol benzoic ester (m/z+ 212) (b) comparison using Labsolutions Shimazu® by NIST.

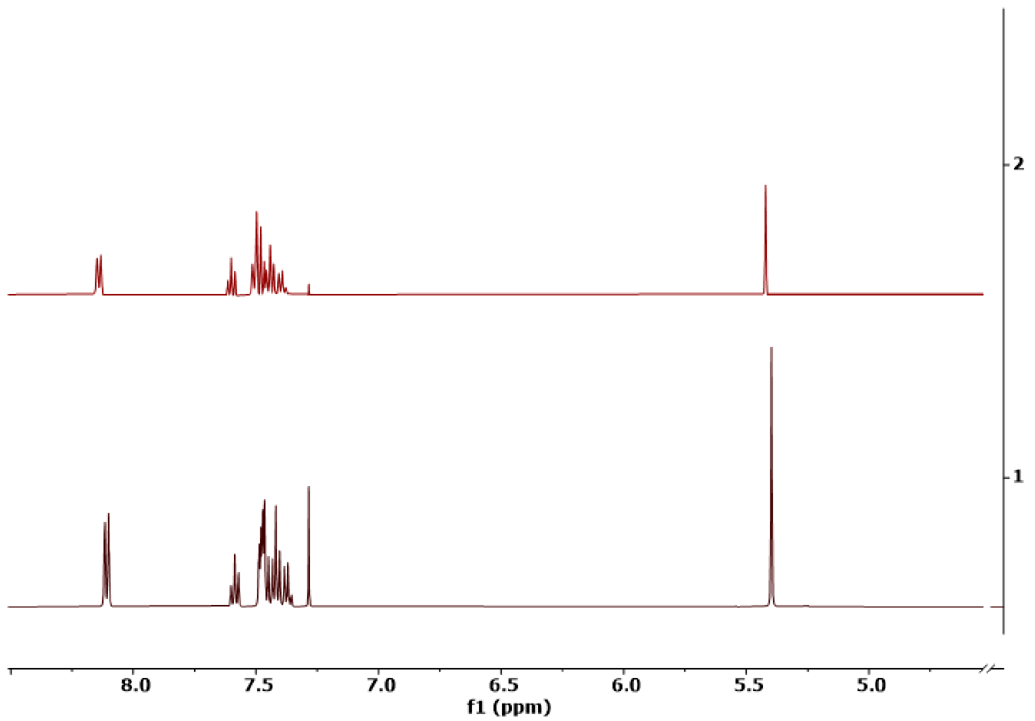

(a)

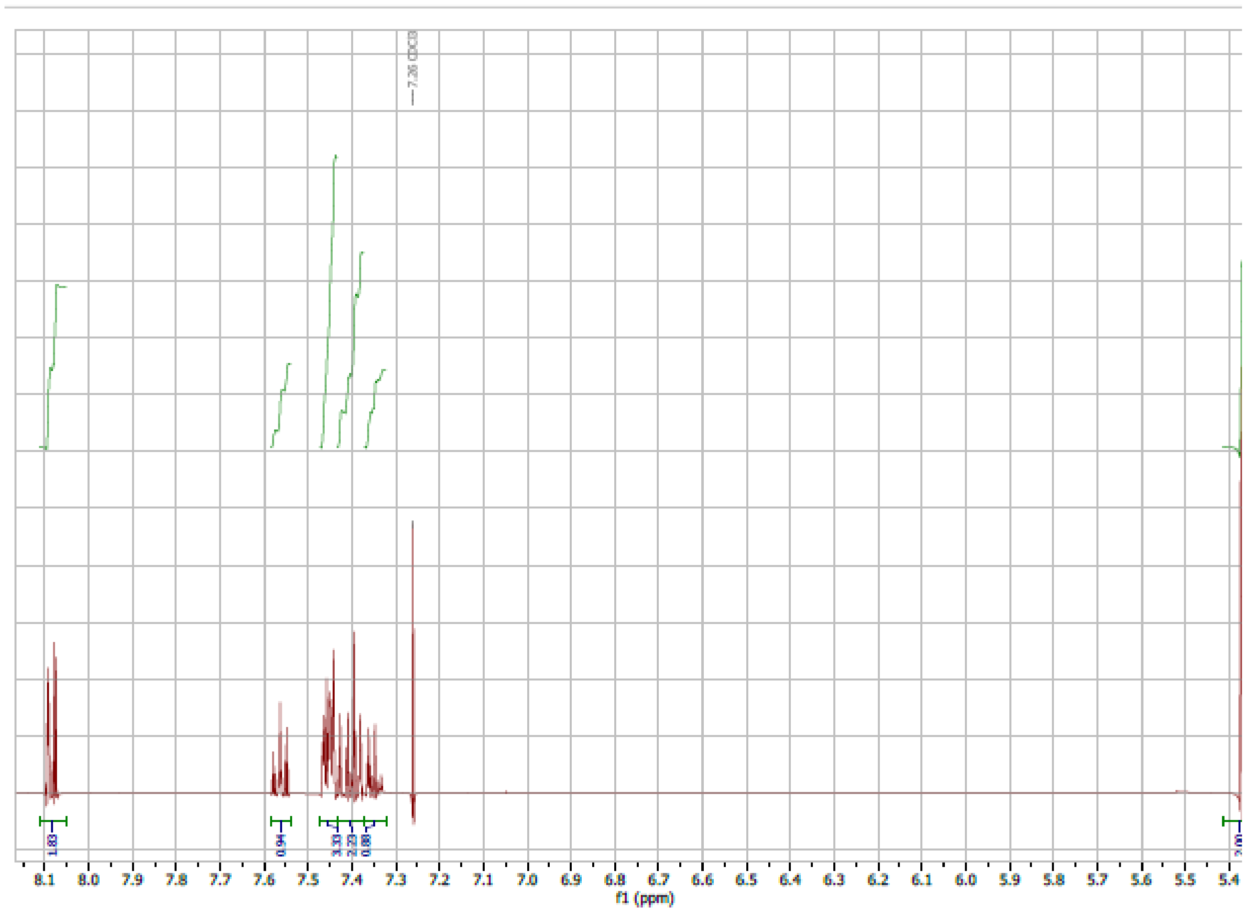

(b)

Figure S3. Nuclear Magnetic Resonance spectrum of (a) stacked ^{1}H NMR HLASFF12 (a natural BB) vs standard reference (BB) (2 vs 1) (b) Integrated ^{1}H NMR of HLASFF12

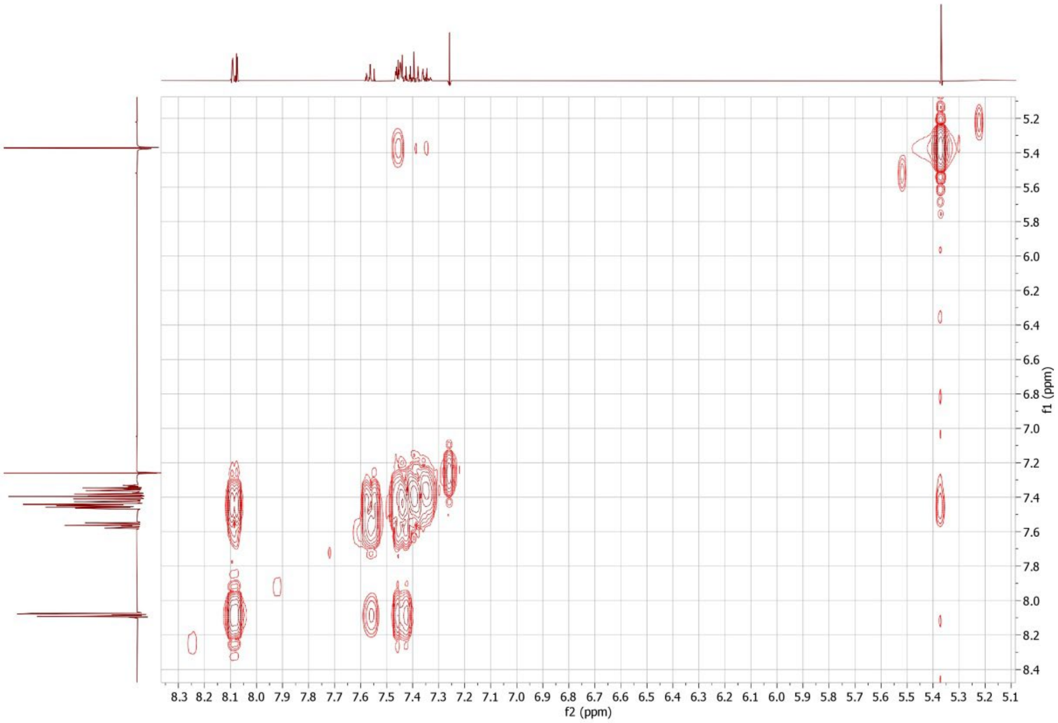

Figure S4: ^{1}H -^{1}H COSY spectrum of benzyl benzoate from *A. smeathamannii*

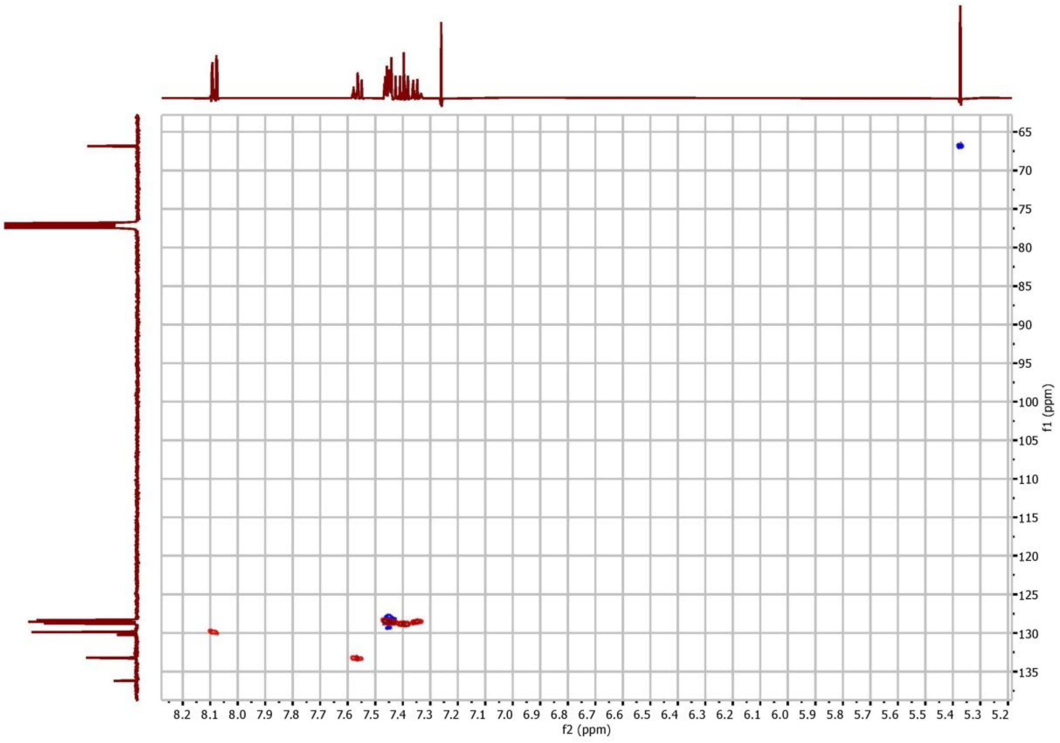

Figure S5: ^{1}H-^{13}C HSQC spectrum of benzyl benzoate from *A. smeathamannii*

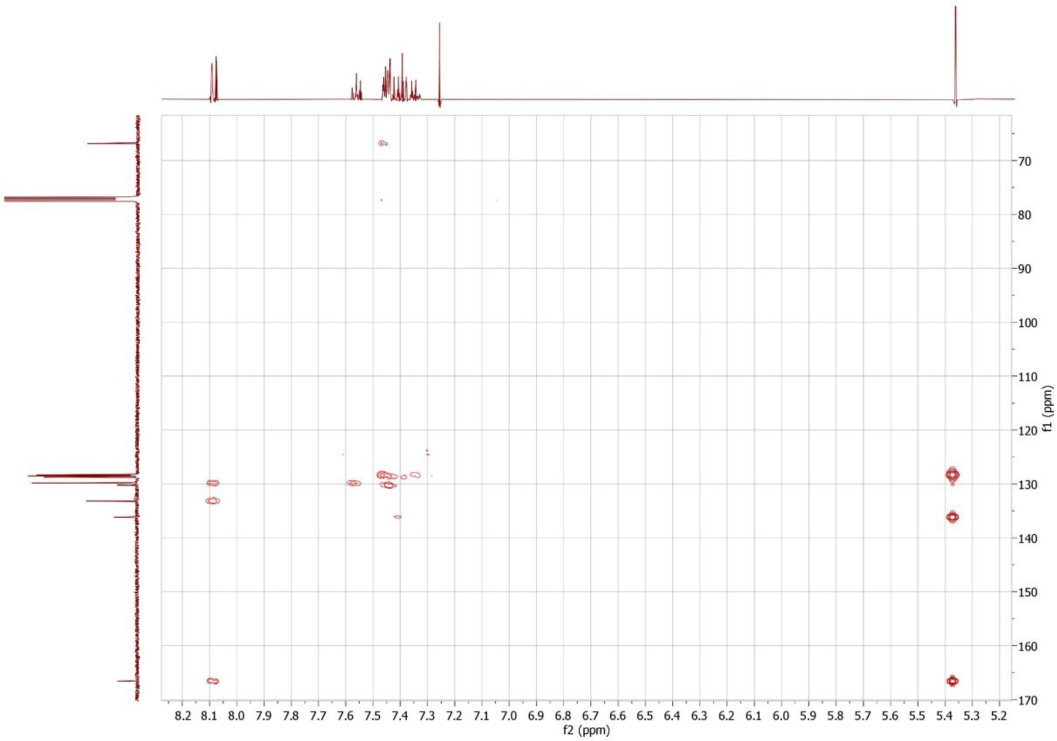

Figure S6: ^{1}H-^{13}C HMBC spectrum of benzyl benzoate from *A. smeathamannii*

02.09.2024 11:36 Chromatogram C:\Clarity Lite\DataFiles\Flavine\Data\Femi-HLASF 11-14 60 MeOH_28.08.2024 15_49_19_039.PRM

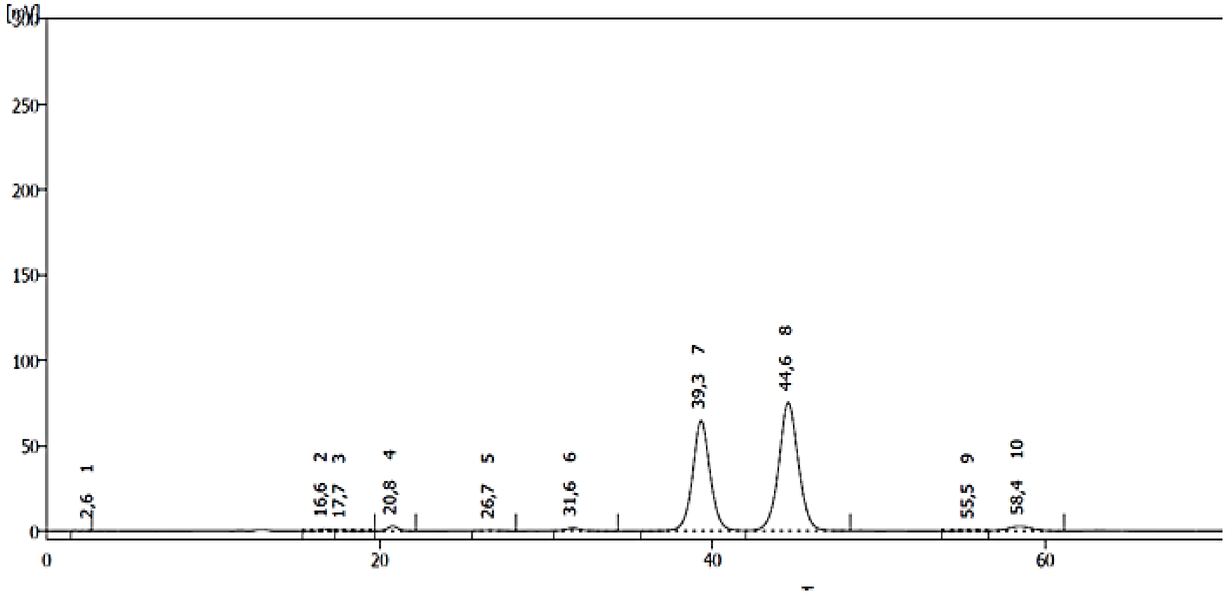

All Signals Result Table (Uncal - Femi-HLASF 11-14 60 MeOH_28.08.2024 15_49_19_039)

	Signal Name	Reten. Time [min]	Area [mV.s]	Height [mV]	Area [%]	Height [%]	W05 [min]
1	Detector 1	2,565	13,246	0,602	0,1	0,4	0,13
2	Detector 1	16,605	52,883	1,244	0,5	0,8	0,65
3	Detector 1	17,705	26,500	0,543	0,2	0,4	0,70
4	Detector 1	20,789	122,021	2,847	1,0	1,9	0,65
5	Detector 1	26,665	19,819	0,316	0,2	0,2	0,92
6	Detector 1	31,591	100,313	1,592	0,9	1,1	0,92
7	Detector 1	39,317	4796,005	64,734	41,0	43,0	1,10
8	Detector 1	44,557	6235,610	75,389	53,4	50,1	1,24
9	Detector 1	55,471	42,620	0,456	0,4	0,3	1,71
10	Detector 1	58,445	276,896	2,688	2,4	1,8	1,57
		All Signals Total	11685,912	150,409	100,0	100,0	

(a)

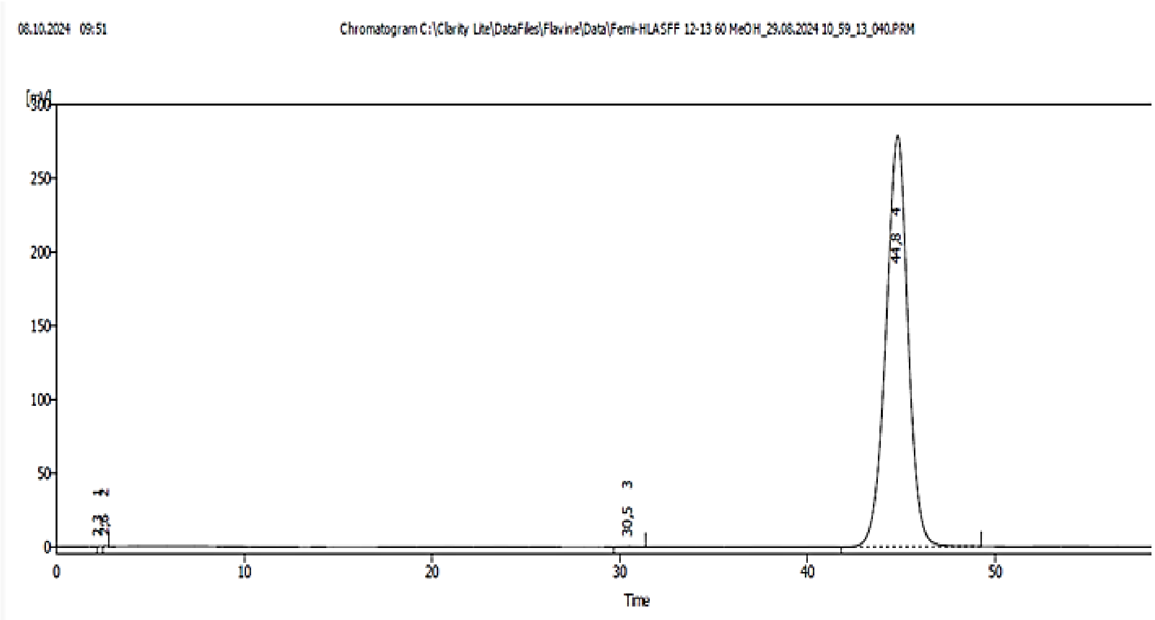

All Signals Result Table (Uncal - Femi-HLASFF 12-13 60 MeOH_29.08.2024 10_59_13_040)

	Signal Name	Reten. Time [min]	Area [mV.s]	Height [mV]	Area [%]	Height [%]	W05 [min]
1	Detector 1	2,307	2,416	0,293	0,0	0,1	0,12
2	Detector 1	2,624	5,149	0,584	0,0	0,2	0,14
3	Detector 1	30,509	10,912	0,220	0,0	0,1	0,81
4	Detector 1	44,805	22754,233	279,031	99,9	99,6	1,23
		All Signals Total	22772,711	280,129	100,0	100,0	

(b)

Figure S7 Spectrometry analysis of *A. smeathmannii* root extract fractions HPLC chromatograms (Aqueous MeOH, 60:40, 280 nm) (a) HLASF 1 - 44 and (b) HLASFF 12

Table S1: NMR Characteristics of HLASFF12

Atom Position	^{13}C Chemical Shift in ppm	^{1}H Chemical Shift in ppm	HH COSY Correlation to H#	HH NOESY Correlation to H#	HMBC Correlation from indexed C to H#
1	166.591				3, 7; 1'
2	130.234				4, 6
3, 7	129.847	8.084	4, 6	1' (w)	5
4, 6	128.742	7.451	3, 7; 5		
5	133.192	7.563	4, 6		3, 7
1'	66.840	5.373	3', 7' (w)	3', 7'; 3, 7(w)	3', 7'
2'	136.157				1'; 4', 6'
3', 7'	128.316	7.436	4', 6'; 1' (w)	1'	1' (s)
4', 6'	128.743	7.396	3', 7'; 5'		
5'	128.392	7.352	4', 6'		
$CDCl_3$	77.160	7.260			

$CDCl_3$: Deuterated chloroform. W: weak bond, S: strong bond
